# Guidelines adherence in the prevention and management of chronic kidney disease in patients with diabetes mellitus on the background of recent European recommendations – a registry-based analysis

**DOI:** 10.1186/s12882-021-02394-y

**Published:** 2021-05-19

**Authors:** Peter Bramlage, Stefanie Lanzinger, Sascha R. Tittel, Eva Hess, Simon Fahrner, Christoph H. J. Heyer, Mathias Friebe, Ivo Buschmann, Thomas Danne, Jochen Seufert, Reinhard W. Holl

**Affiliations:** 1 Institute for Pharmacology and Preventive Medicine, Bahnhofstrasse 20, 49661 Cloppenburg, Germany; 2 Institute for Epidemiology and Medical Biometry, ZIBMT, University of Ulm, Ulm, Germany; 3 German Center for Diabetes Research (DZD), Munich-Neuherberg, Germany; 4 Specialized Diabetes Practice Dres. Hess, Worms, Germany; 5 Medical Clinic, SRH Clinic Sigmaringen, Pfullendorf, Germany; 6 Specialized Diabetes Practice Viersen, Viersen, Germany; 7 Protestant Hospital, Oberhausen, Germany; 8 DAZB (German Angiology Center Brandenburg/Berlin), Medical School Brandenburg (MHB) & Faculty of Health Science FGW, Brandenburg, Germany; 9 European Foundation for Vascular Medicine (EFVM), Brandenburg Havel, Germany; 10 Kinderkrankenhaus auf der Bult, Diabetes Centre for Children and Adolescents, Hannover, Germany; 11 Division of Endocrinology and Diabetology, Department of Medicine II, Medical Center, Faculty of Medicine, University of Freiburg, Freiburg, Germany

**Keywords:** diabetes, hypertension, chronic kidney disease, glomerular filtration rate, albuminuria, diagnostics, pharmacotherapy

## Abstract

**Background:**

Recent European Society of Cardiology (ESC)/European Association for the Study of Diabetes (EASD) guidelines provide recommendations for detecting and treating chronic kidney disease (CKD) in diabetic patients. We compared clinical practice with guidelines to determine areas for improvement.

**Methods:**

German database analysis of 675,628 patients with type 1 or type 2 diabetes, with 134,395 included in this analysis. Data were compared with ESC/EASD recommendations.

**Results:**

This analysis included 17,649 and 116,747 patients with type 1 and type 2 diabetes, respectively. The analysis showed that 44.1 and 49.1 % patients with type 1 and type 2 diabetes, respectively, were annually screened for CKD. Despite anti-diabetic treatment, only 27.2 % patients with type 1 and 43.5 % patients with type 2 achieved a target HbA1c of < 7.0 %. Use of sodium-glucose transport protein 2 inhibitors (1.5 % type 1/8.7 % type 2 diabetes) and glucagon-like peptide-1 receptor agonists (0.6 % type 1/5.2 % type 2 diabetes) was limited. Hypertension was controlled according to guidelines in 41.1 and 67.7 % patients aged 18–65 years with type 1 and 2 diabetes, respectively, (62.4 vs. 68.4 % in patients > 65 years). Renin angiotensin aldosterone inhibitors were used in 24.0 and 40.9 % patients with type 1 diabetes (micro- vs. macroalbuminuria) and 39.9 and 47.7 %, respectively, in type 2 diabetes.

**Conclusions:**

Data indicate there is room for improvement in caring for diabetic patients with respect to renal disease diagnosis and treatment. While specific and potentially clinically justified reasons for non-compliance exist, the data may serve well for a critical appraisal of clinical practice decisions.

## Background

Early detection and treatment of chronic kidney disease (CKD) in patients with diabetes may prevent or delay the development of end-stage renal disease (ESRD), as well as subsequent morbidity and mortality. The Kidney Disease Outcomes Quality Initiative (KDOQI) of the National Kidney Foundation has recommended the use of estimating equations for glomerular filtration rate (GFR) on the basis of serum creatinine determinations and urinary albumin-to-creatinine ratio (UACR) since 2011 [1, 2].

Screening recommendations are essentially unaltered since 2011 [3, 4]. The 2019 guidelines of the European Society of Cardiology (ESC)/European Association for the Study of Diabetes (EASD) [3] recommend that patients with chronic kidney disease undergo annual spot UACR, serum creatinine and estimated GFR (eGFR) evaluations (recommendation class I, level of evidence A) along with a number of specific recommendations for the treatment of affected patients. Particular attention must be given to tight glucose and blood pressure control: It is recommended to target an HbA1c < 7.0 % (or < 53 mmol/mol) to decrease microvascular complications (recommendation class I, level of evidence A). Blood pressure should be lowered to 130 mmHg systolic blood pressure (SBP) or lower (but not < 120 mmHg), while a SBP between 130 and 139 mmHg is recommended for patients > 65 years (recommendation class I, level of evidence A). Diabetic patients with hypertension should be treated with renin-angiotensin-aldosterone system (RAAS)-blocking agents (angiotensin converting enzyme inhibitor [ACEi]/angiotensin receptor blocker [ARBs]) especially when microalbuminuria, macroalbuminuria/proteinuria, or left ventricular hypertrophy (LVH) is present (recommendation class I, level of evidence A). Treatment with sodium-glucose transport protein 2 inhibitors ([SGLT-2i] empagliflozin, canagliflozin, or dapagliflozin) is advised in patients with an eGFR 30 to < 90 mL/min/1.73 m^2^) (recommendation class I, level of evidence B) and glucagon-like peptide-1 receptor agonist ([GLP-1Ras] liraglutide, semaglutide) in patients if eGFR is > 30 mL/min/1.73m^2^ (recommendation class IIa, level of evidence B).

The aim of our study, using a large combined database of 675,628 patients with diabetes treated in Germany, [5, 6] was to determine rates of adherence to treatment guidelines, gain a better understanding of the medications used to treat diabetes in Germany, determine areas for the improvement in the screening of diabetic patients, and assess the use of potentially beneficial treatment strategies.

## Methods

### Study design and data sources

This analysis used combined data from the Diabetes Patienten Verlaufsdokumentation (DPV) and Diabetes Versorgungsevaluation (DIVE) registries. Their design has been described previously [7–9]. In short, the DPV initiative collects data on patients with diabetes mellitus from centres predominantly in Germany. The diagnosis of diabetes type, selection of medication used, types of assessment conducted, and information recorded in the database are made by the patient’s clinician. Data are collected every 6 months using DPV software and the anonymized data are sent to the University of Ulm for aggregation into the database. The DPV initiative was established in 1995, approved by the University of Ulm ethics committee, and data collection approved by local review boards. The DIVE registry was established in 2011. Consecutive patients with diabetes mellitus, regardless of their disease stage, were enrolled from centres across the country, and continue to be followed up. The DIVE protocol was approved by the ethics committee of the Medical School of Hannover, and all patients included in the DIVE registry provided written informed consent.

### Patient selection

**Fig. 1:**
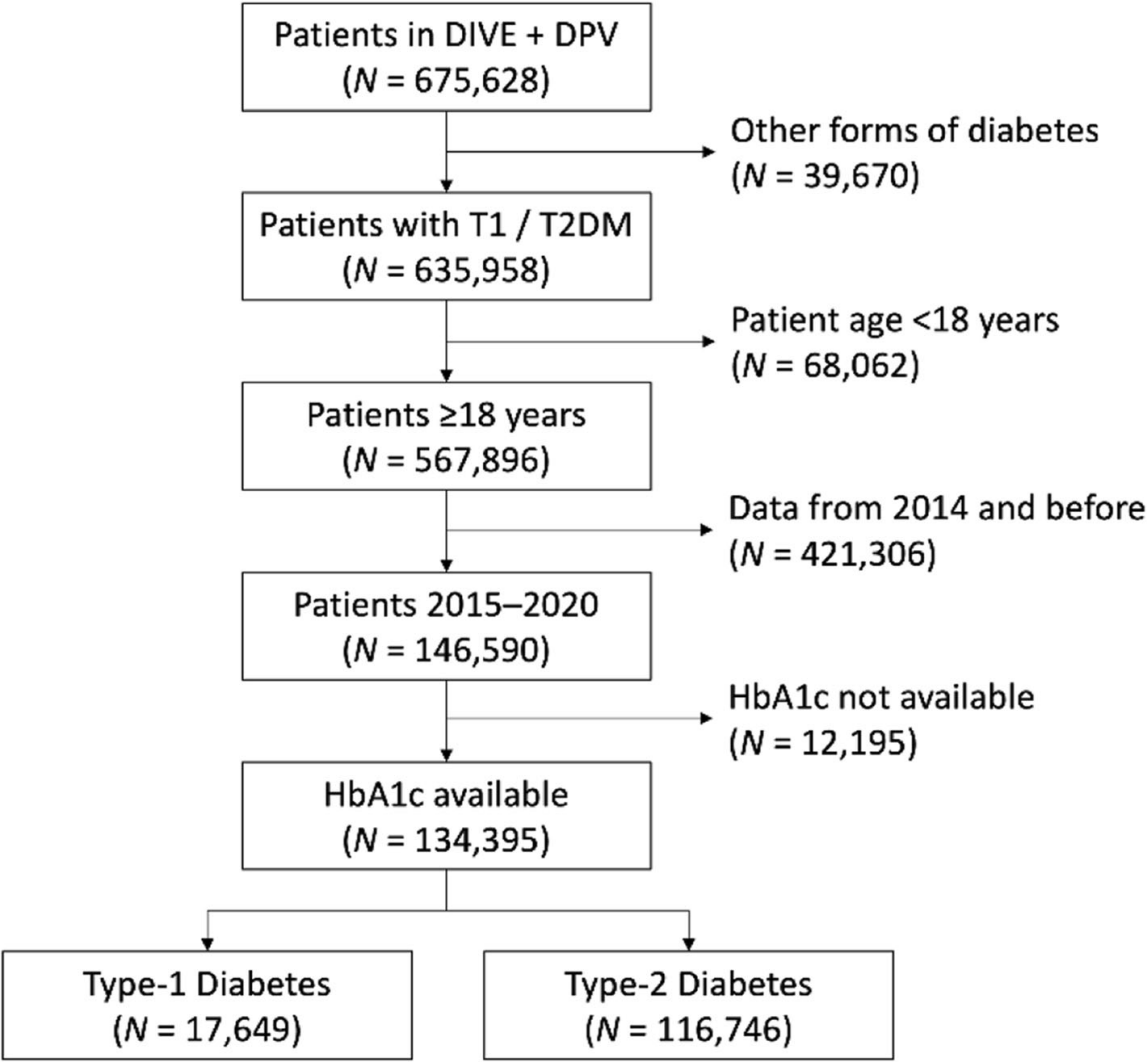
Patient population

From a total of 144 DIVE and 503 DPV centres from Germany, 416 were included in the present analysis based on their provision of patient data eligible for the analysis (Fig. 1). The last patient considered for this analysis was from September 2020. They were included in the current analysis if they had type 1 or type 2 diabetes, were at least 18 years old, and were confined to those whose details were first entered in the database between 2015 and 2020, and had HbA1c information available.

### Definitions

GFR was estimated based on the Chronic Kidney Disease Epidemiology Collaboration (CKD-EPI) formula. Microalbuminuria was defined as an UACR between 30 and 300 mg/g or an albumin excretion of 30 to 300 mg/L, macroalbuminuria/proteinuria as an UACR of at least 300 mg/g or an albumin excretion of at least 300 mg/L. CKD was defined as either an eGFR < 60 ml/min/1.73 m^2^ and/or at least microalbuminuria and/or kidney transplantation/dialysis. Hypertension was defined as an SBP > 130 mmHg systolic in patients ≤ 65 years and > 140 mmHg in patients > 65 years and/or antihypertensive treatment. Controlled hypertension was defined as a blood pressure ≤ 130 mmHg but not below 120 mmHg in patients ≤ 65 years and a blood pressure 130–139 mmHg in patients > 65 years.

### Statistics

**Fig. 2:**
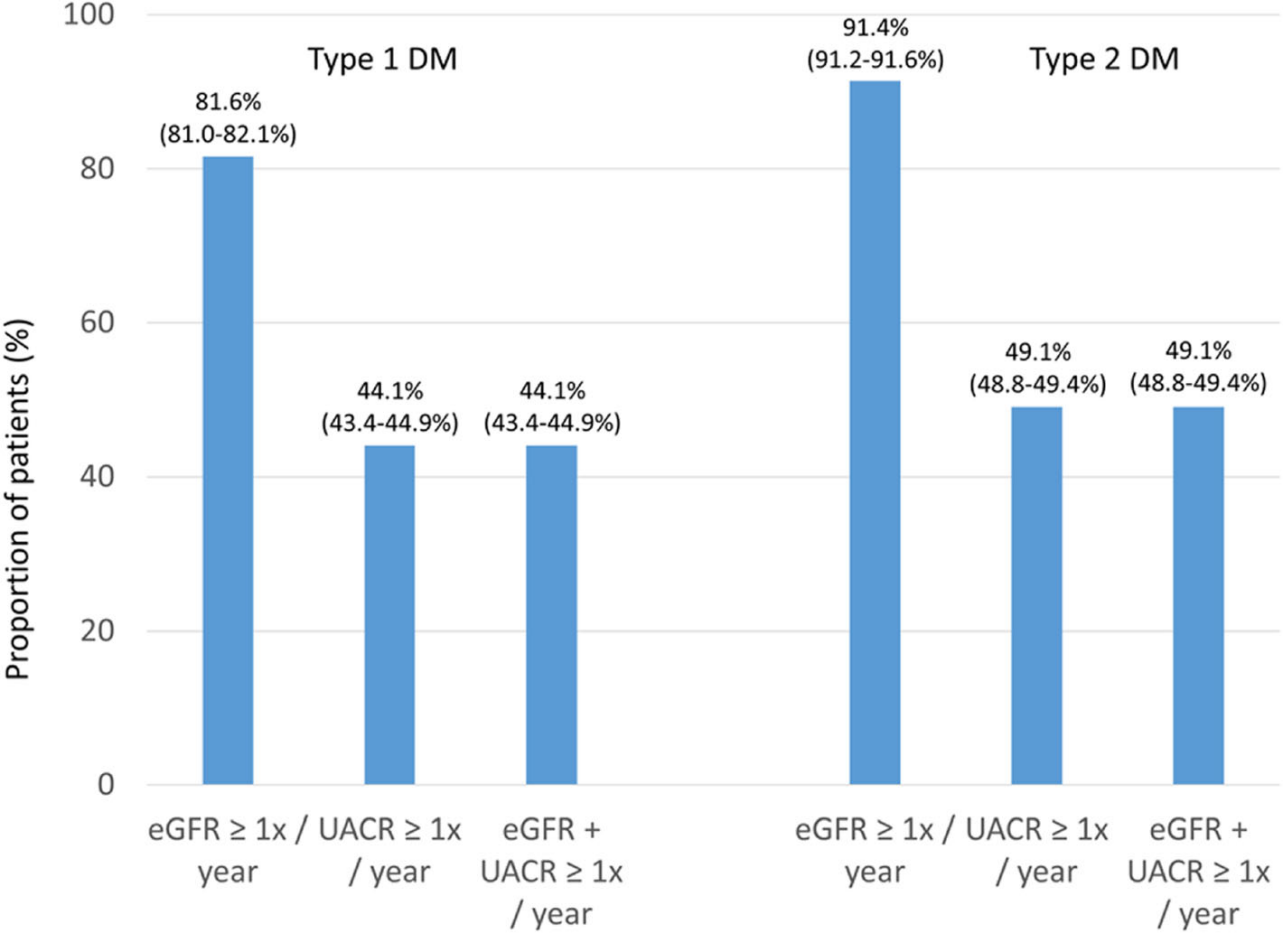
Proportion of patients undergoing guideline-recommended GFR/UACR assessments. Legend: It is recommended that patients with diabetes are screened annually for kidney disease by assessment of eGFR and urinary albumin:creatinine ratio [3]. Values are percent with 95 % CIs; values are from the last treatment year.

**Fig. 3:**
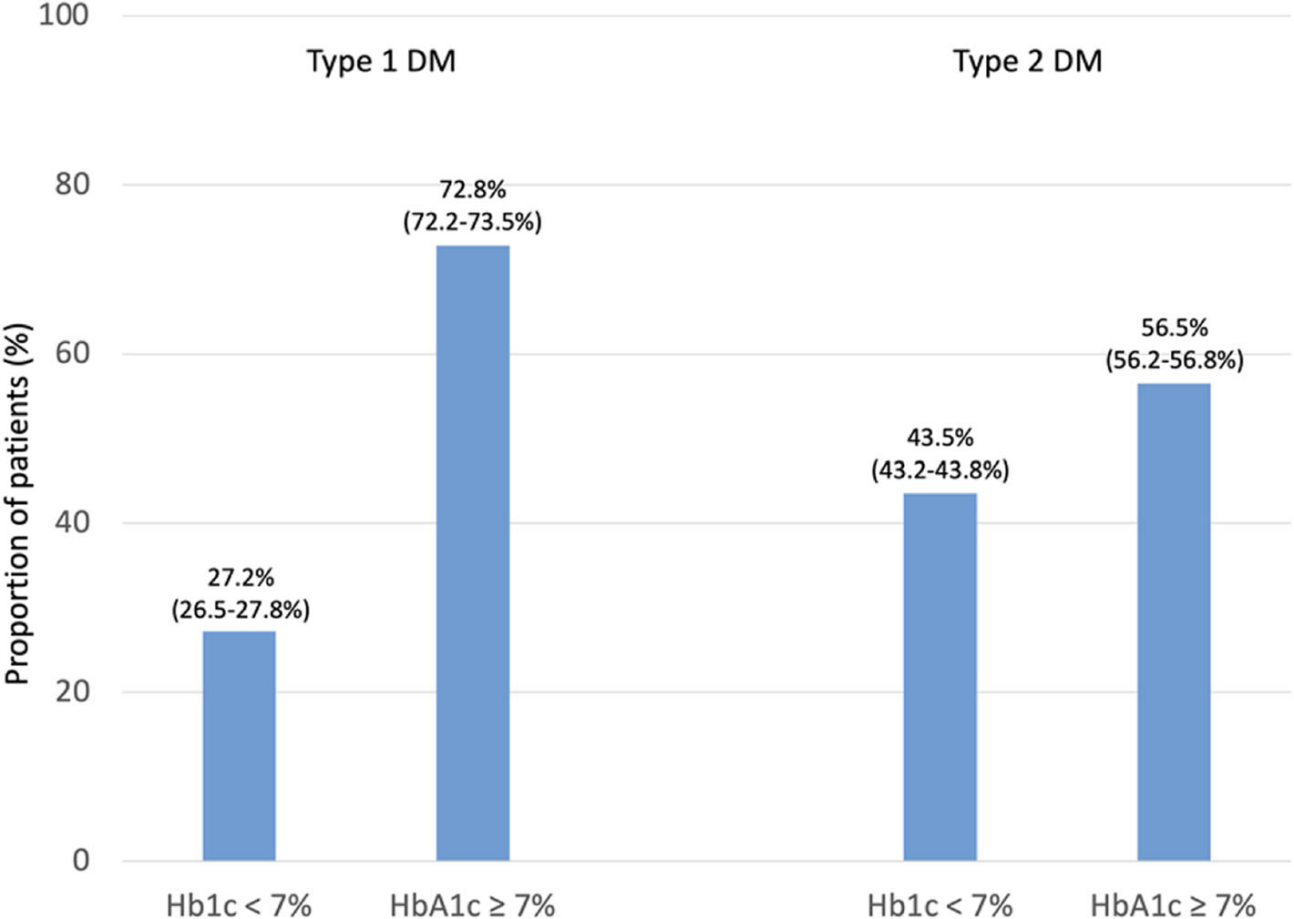
Proportion of patients achieving HbA1c targets as per guideline recommendation. Legend: Tight glucose control, targeting HbA1c < 7.0 % (or < 53 mmol/mol), is recommended to decrease microvascular complications in patients with diabetes [3]. Values are percent with 95 % CIs.

**Fig. 4:**
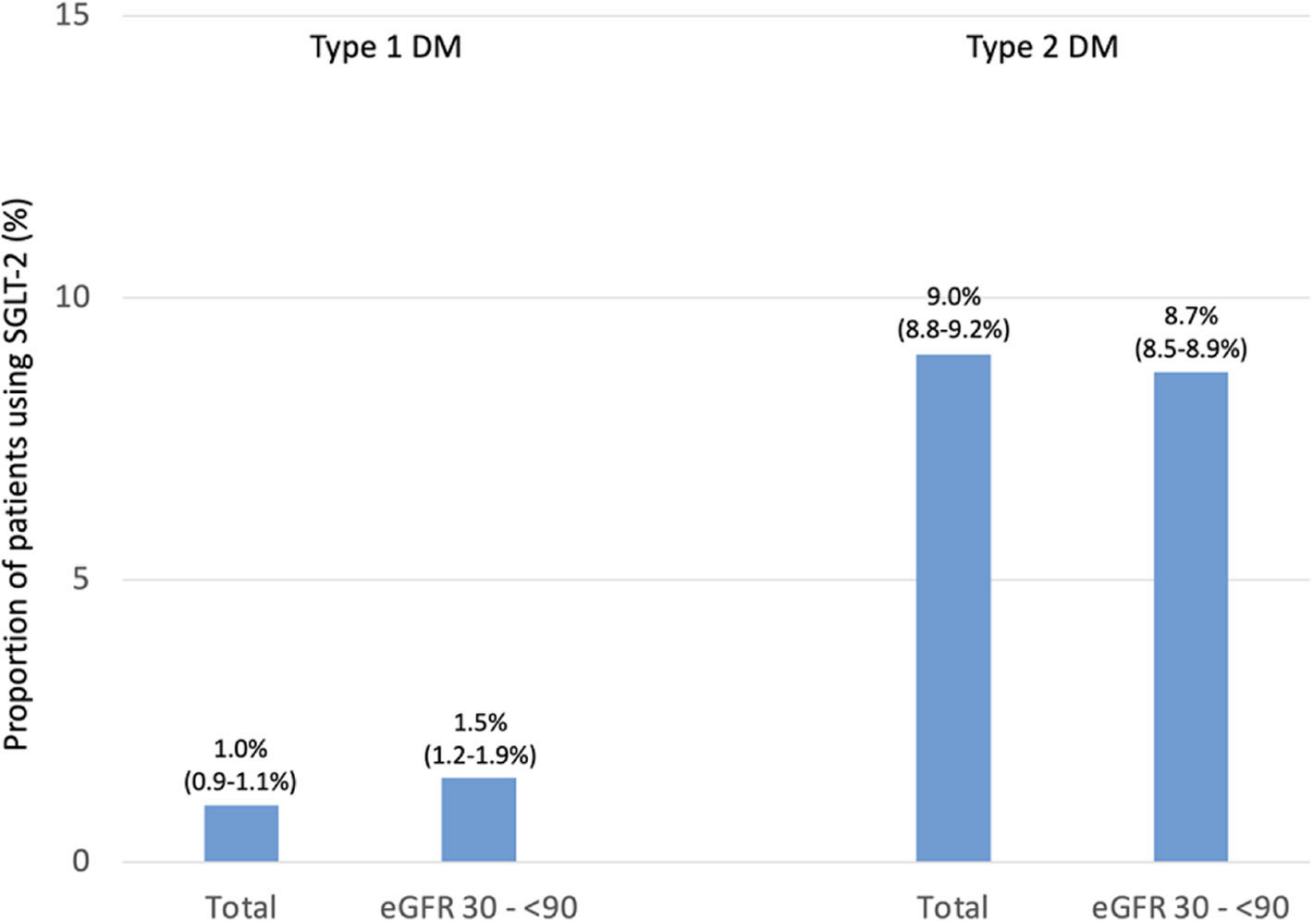
Proportion of patients using SGLT-2 as per guideline recommendation.  Legend: Treatment with an SGLT2 inhibitor (empagliflozin, canagliflozin or dapagliflozin) is associated with a lower risk of renal endpoints and is recommended* if eGFR is 30 to < 90 mL/min/1.73 m^2^) [3]. Analyses restricted to the last treatment year; values are percent with 95 % CIs; *Although evidence is very limited for patients with T1DM

**Fig. 5:**
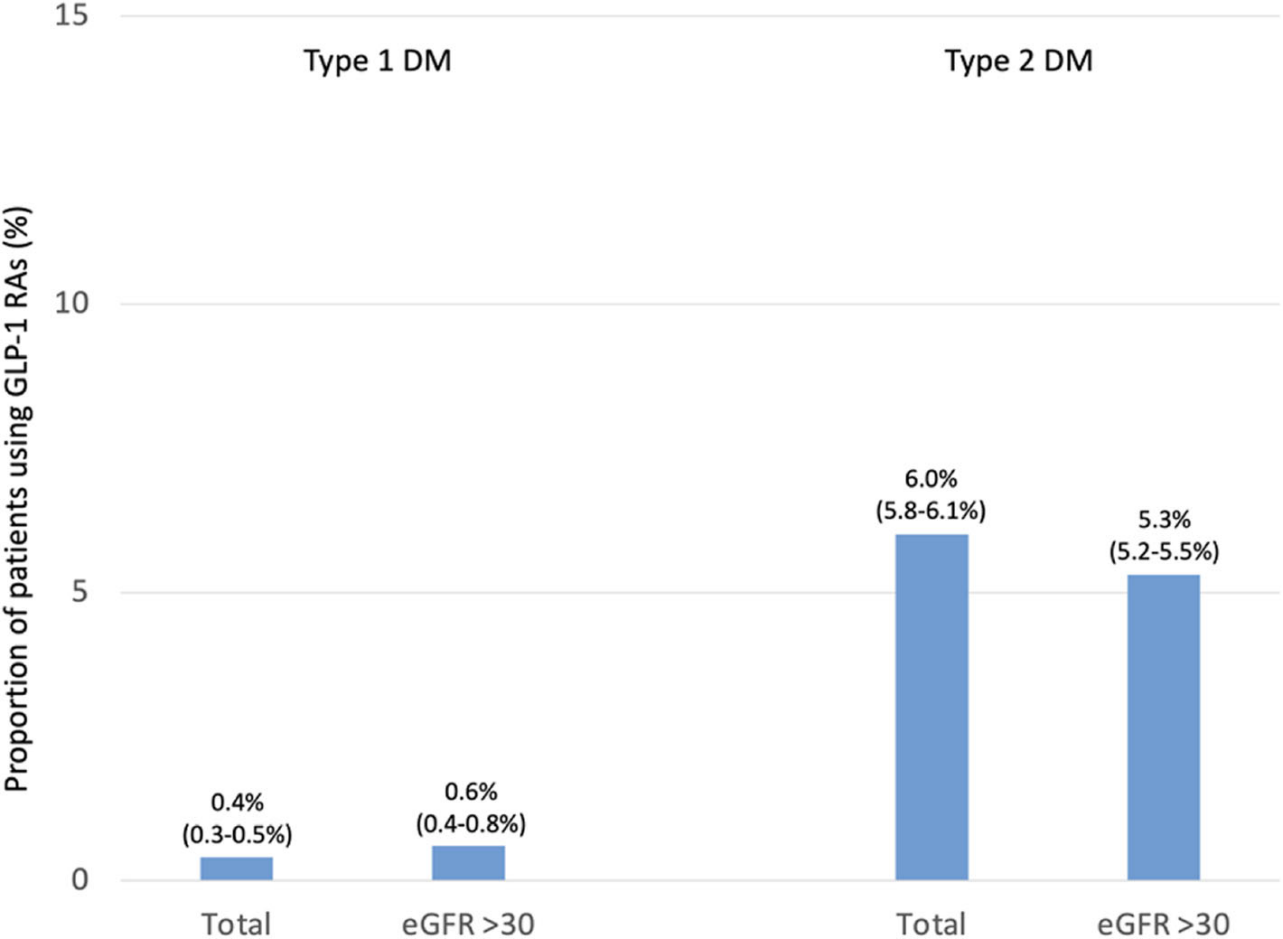
Proportion of patients using GLP-1 RAs as per guideline recommendation. Legend: Treatment with the GLP1-RAs liraglutide and semaglutide is associated with a lower risk of renal endpoints, and should be considered* for DM treatment if eGFR is > 30 mL/min/1.73 m^2^ [3]. Analyses restricted to the last treatment year; values are percent with 95 % CIs; *Although evidence is not available for patients with T1DM

**Fig. 6:**
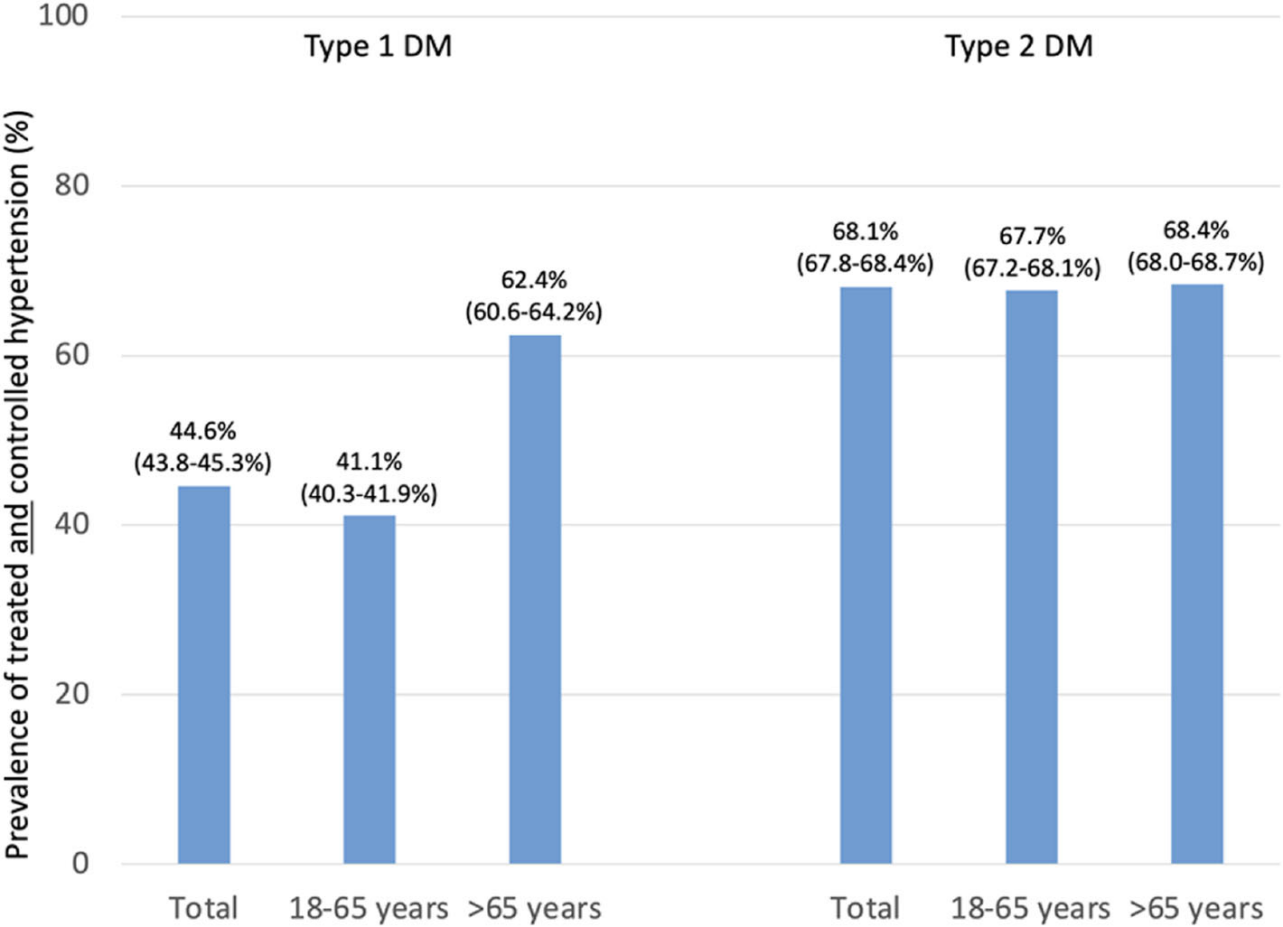
Prevalence of treated and controlled hypertension. Legend: It is recommended that patients with hypertension (> 130 mmHg systolic and/or antihypertensive drug use) and diabetes are treated in an individualised manner, SBP to 130 mmHg and < 130 mmHg if tolerated, but not < 120 mmHg. In older people (aged > 65 years) with hypertension (> 140 mmHg systolic and/or antihypertensive treatment) the SBP goal is to a range of 130–139 mmHg [3]. Values are percent with 95 % CIs

**Fig. 7:**
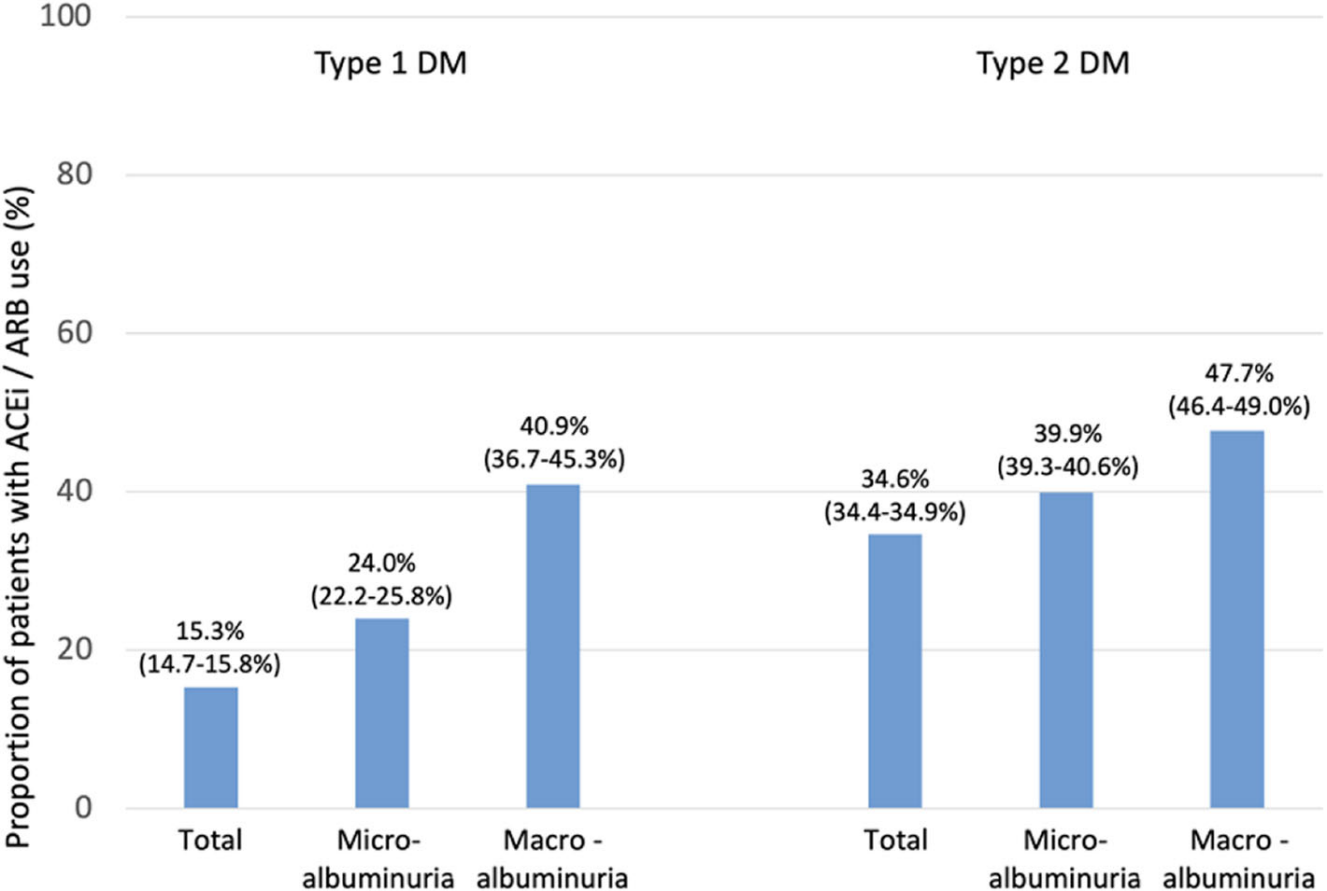
Proportion of patients using ACEi/ARBs as per guideline recommendation. Legend: A RAAS blocker (ACEi or ARB) is recommended for the treatment of hypertension in patients with diabetes, particularly in the presence of microalbuminuria, proteinuria (macroalbuminuria*), or LVH** [3]. Values are percent with 95 % CIs; *Proteinuria was replaced by macroalbuminuria as this was generally determined; **LVH is not available in the DIVE/DPV dataset

Data from all patients were combined and analyzed as a single data set. Categorical variables are presented as percentages. Continuous variables are presented as means with standard deviations. Patient characteristics were described by the type of diabetes. Estimates for the proportion of patients (%) reported in Figs. 1, 2, 3, 4, 5, 6 and 7 were provided together with 95 % confidence intervals (CI) for the last treatment year. Statistical analysis was performed using Statistical Analysis System, version 9.4 (SAS, North Carolina, USA).

## Results

The dataset comprised 675,978 patients (Fig. 1), of which 134,395 had either type 1 or type 2 diabetes, were 18 years or older, had in- or outpatient visits between 2015 and 2020 and at least one HbA1c value available. Of these, 17,649 had type 1 and 116,746 had type 2 diabetes.

### Patient characteristics

Patients with type 1 diabetes (Table 1) had a mean age of 45.0 years, 53.7 % were male, the mean diabetes duration was 15.9 years, the mean body mass index (BMI) 25.9 kg/m^2^, 11.9 % had a history of cardiovascular disease and the mean HbA1c was 8.3 % (67.7 mmol/mol). Their eGFR was 92.7 mL/min/1.73 m^2^, 20.6 % had microalbuminuria, 5.1 % macroalbuminuria and the mean serum potassium was 5.5 mEq/L.

Patients with type 2 diabetes had a mean age of 67.8 years, 55.4 % were male, the mean diabetes duration was 10.0 years, the mean BMI 31.3 kg/m^2^, 29.1 % had a history of cardiovascular disease and the mean HbA1c was 7.8 % (61.3 mmol/mol). Their eGFR was 68.3 mL/min/1.73 m^2^, 30.6 % had microalbuminuria, 9.1 % macroalbuminuria and the mean serum potassium was 5.3 mEq/L.

Beyond obvious differences between patients with type 1 and type 2 diabetes such as age, BMI and duration of diabetes, patients with type 2 diabetes had a higher mean blood pressure, a higher comorbidity burden and a lower HbA1c. Differences in the eGFR were substantial with an absolute difference of + 24.4 mL/min/1.73 m^2^ for type 1 vs. type 2 diabetes, and an absolute difference in the rate of microalbuminuria of -10 %. Drugs that are considered to have an effect on renal function were more widely prescribed in type 2 diabetes such as ACEi (22.9 vs. 10.2 %), ARBs (14.0 vs. 6.2 %), mineralocorticoid receptor antagonists ([MRAs] 2.5 vs. 0.5 %) and SGLT-2is (9.0 vs. 1.0 %).

**Table 1:** Patient characteristics

	Type 1 DM (*n* = 17,649)		Type 2 DM (*n* = 116,746)	
	N avail	Mean ± SD or %	N avail	Mean ± SD or %
Age, years	17,649	45.0 ± 18.4	116,746	67.8 ± 13.8
Gender, male	17,649	53.7	116,746	55.4
BMI, kg/m^2^	16,708	25.9 ± 5.6	107,490	31.3 ± 7.1
Duration of diabetes, years	17,649	15.9 ± 15.3	116,746	10.0 ± 9.4
HbA1c, %	17,649	8.3 ± 2.1	116,746	7.8 ± 2.0
mmol/mol	17,649	67.7 ± 23.2	116,746	61.3 ± 22.2
Systolic blood pressure, mmHg	16,717	129.2 ± 17.0	111,354	135.2 ± 18.6
Diastolic blood pressure, mmHg	16,644	77.1 ± 10.2	110,936	77.4 ± 11.1
Diabetic retinopathy	6,355	17.7	36,681	8.7
Diabetic neuropathy	17,649	27.2	116,746	37.2
Lipid metabolism disorders	10,880	41.8	77,081	53.4
History of CV disease	17,649	11.9	116,746	29.3
Coronary artery disease	17,649	6.0	116,746	16.8
Peripheral arterial disease	17,649	5.7	116,746	12.1
Heart failure	17,649	2.9	116,746	12.4
Myocardial infarction	17,649	2.7	116,746	7.4
Stroke	17,649	2.9	116,746	7.5
PCI/Stent	17,649	0.5	116,746	1.6
eGFR (CKD-EPI), mL/min/1.73 m^2^	14,396	92.7 ± 28.6	106,692	68.3 ± 27.1
≥ 60	14,396	86.4	106,692	61.4
45 to 59	14,396	6.3	106,692	16.1
30 to 44	14,396	3.7	106,692	13.1
15–29	14,396	2.0	106,692	7.2
< 15	14,396	1.6	106,692	2.2
Albuminuria determination				
Albumin	9,917	56.2	64,403	55.2
Albumin:Creatine ratio	7,790	44.1	57,321	49.1
Albuminuria				
Normoalbuminuria	10,630	79.4	69,839	69.4
Microalbuminuria	10,630	20.6	68,839	30.6
Macroalbuminuria	9,939	5.1	64,472	9.1
Serum potassium, mEq/L, mean (SD)	4,028	5.5 ± 3.8	27,115	5.3 ± 3.6
< 3.5 mEq/L	4,028	5.0	27,115	5.4
3.5–5.0 mEq/L	4,028	78.5	27,115	78.7
> 5.0–5.5 mEq/L	4,028	4.9	27,115	5.9
> 5.5 mEq/L	4,028	11.6	27,115	10.0
RAS-blockers				
ACEi	17,649	10.2	116,746	22.9
ARBs	16,388	6.2	116,746	14.0
ACEi + ARBs*	17,649	0.7	116,746	1.3
MRA	17,649	0.5	116,746	2.5
GLP1-RA / SGLT-2i use				
GLP-1 RA	17,649	0.4	116,746	6.0
SGLT-2i	17,649	1.0	116,746	9.0

Legend: *ACEi* angiotensin converting enzyme inhibitor; *ARB* angiotensin receptor blocker; *BMI* body mass index; *CKD-EPI* Chronic Kidney Disease Epidemiology Collaboration; *SD* standard deviation; *DPP4* dipeptidyl peptidase-4; *eGFR* estimated glomerular filtration rate; *GLP-1* glucagon-like peptide-1; *HDL* high density lipoprotein; *LDL* low density lipoprotein; *MRAs* mineralocorticoid receptor antagonists; *PCI* percutaneous coronary intervention; *SGLT-2*, sodium-glucose transport protein-2; *Patients are part of ACEi and ARB rows as well; **Non-insulin antidiabetic therapy in T1DM needs to be interpreted with caution, as they may include patients with latent autoimmune diabetes in adults (LADA)

### CKD screening

The ESC/EASD guidelines recommend that patients with diabetes are screened annually for the presence of kidney disease by the assessment of eGFR and UACR (recommendation class I, level of evidence A) [3]. In DIVE/DPV a regular (≥ 1x/year) determination of the eGFR (Fig. 2) was more frequent in type 2 (91.4 %) than in type 1 diabetes (81.6 %). The frequency of regular urinary albumin:creatinine determinations was comparable in type 1 (44.1 %) and type 2 diabetes (49.1 %). The proportion of patients who received at least one eGFR and one albumin:creatinine determination was comparable in type 1 (44.1 %) and type 2 diabetes (49.1 %).

### Glucose control

Tight glucose control, targeting HbA1c < 7.0 % (or < 53 mmol/mol), is recommended by the ESC/EASD guideline [3] to decrease microvascular complications in patients with diabetes (recommendation class I, level of evidence A). In our dataset, 27.2 % of patients with type 1 (Fig. 3) and 43.5 % of patients with type 2 diabetes had an HbA1c that was in line with the above-mentioned recommendations.

Treatment with an SGLT-2i (empagliflozin, canagliflozin or dapagliflozin) is recommended as it is associated with a lower risk of renal endpoints [3] if eGFR is 30 to < 90 mL/min/1.73 m^2^ (recommendation class I, level of evidence B). In DIVE/DPV, treatment with SGLT-2i was not abundant with 1.5 % of patients with type 1 and 8.7 % of patients with type 2 diabetes receiving them (Fig. 4). As opposed to the guidelines, rates did not change for the patients within the eGFR range 30 to < 90 mL/min/1.73 m^2^. Treatment was generally initiated after the diagnosis of CKD. Use of SGLT-2 inhibitors was not abundant in type 1 diabetes and patients receiving them were characterized by a higher mean age (54.6 vs. 44.9 years), higher proportion of males (62.1 vs. 53.6 %), a higher BMI (28.9 vs. 25.8 kg/m^2^) and a higher prevalence of CVD (25.3 vs. 11.8 %).

Treatment with the GLP1-RAs liraglutide and semaglutide should be considered [3] if eGFR is > 30 mL/min/1.73 m^2^ as it is associated with a lower risk of renal endpoints (recommendation class IIa, level of evidence B). Treatment with GLP-1 RAs was not abundant with 0.6 % of patients with type 1 and 5.3 % of patients with type 2 diabetes receiving them (Fig. 5). Rates were comparable in patients with an eGFR > 30 mL/min/1.73 m^2^, not in line with guideline recommendations. Treatment was again generally initiated after the diagnosis of CKD. Patients with type 1 diabetes receiving GLP-1RAs were characterized by a higher age (52.8 vs. 44.9 years), lower proportion of males (47.8 vs. 53.8 %), a higher BMI (34.8 vs. 25.8 kg/m^2^) and a higher prevalence of CVD (26.9 vs. 11.9 %)

### Blood pressure control

The guidelines [3] recommend that patients with hypertension and diabetes are treated in an individualized manner, SBP to 130 mmHg and < 130 mmHg if tolerated, but not < 120 mmHg. In patients > 65 years the SBP goal lies within a range of 130–139 mmHg (recommendation class I, level of evidence A). In the DIVE/DPV registry, hypertension control was 41.1 % in type 1 and 67.7 % in type 2 diabetes in patients 18–65 years (Fig. 6). It was 62.4 and 68.4 % in those > 65 years, respectively.

RAAS blockers (ACEi or ARB) are recommended [3] for the treatment of hypertension in patients with diabetes mellitus, particularly in the presence of microalbuminuria, proteinuria or LVH (recommendation class I, level of evidence A). LVH was unfortunately not available in the current dataset. ACEi or ARB use was increased in patients with macroalbuminuria/proteinuria (40.9 %) and microalbuminuria (24.0 %) compared with the total population of patients with type 1 diabetes (15.3 %) (Fig. 7). The same was true for patients with type 2 diabetes (47.7 % and 39.0 vs. 34.6 %). ACEi/ARB use was also higher in patients with a GFR < 60 mL/min/1.73 m^2^ (35.9 % type 1; 41.0 % type 2 diabetes) than in patients with a GFR ≥ 60 mL/min/1.73 m^2^ (14.3 and 33.5 %, respectively). ACEi/ARB use was also higher in patients with CKD (26.2 % type 1; 39.5 % type 2 diabetes) than in patients without (12.7 and 31.7 %, respectively).

## Discussion

This database analysis shows that (1) about every other patient with diabetes is screened for the presence of CKD annually; (2) less than half of patients achieve a target HbA1c of < 7.0 % (or < 53 mmol/mol); recommended use of SGLT-2i and GLP1-RA is well below 10 %; (3) hypertension is controlled in about two-thirds of patients with lower rates depending on age and respective SBP thresholds; RAAS blockers are used in about half of the patients. Taken together there appears to be room for improvement with respect to the renal aspects of diabetes care to prevent kidney-associated morbidity and mortality. It is important to understand guideline compliance because adherence to treatment guidelines is associated with improved clinical outcomes in patients with type 2 diabetes, better glycaemic and blood pressure control, and it has the potential to reduce the risk of developing CKD/ESRD through comprehensive patient assessment [10, 11].

### Chronic Kidney Disease Screening

Renal involvement in diabetes is common and CKD significantly increases the risk of atrial fibrillation in patients with diabetes [12]. It is essential, therefore, that kidney involvement is detected as early as possible. The ESC/EASD recommendations to screen patients annually for kidney disease using eGFR and UACR has also been recommended by the 2020 American Diabetes Association (ADA) [4] and the 2013 Kidney Disease: Improving Global Outcomes (KDIGO) Clinical Practice Guideline [13]. The recent 2020 KDIGO guidelines [14] supports this approach in a more general sense as they recommend multifactorial diabetes management with yearly assessment of urine albumin excretion and eGFR, but refer to primary care and endocrinology society guidelines for further details.

Only about half of the patients with diabetes, irrespective of diabetes type, were screened annually in DIVE/DPV, which appears low. The data are, however, not far off from those of Hagnas et al., [15] which investigated CKD-screening in Finnish primary healthcare, where 60.2 % of 5,112 patients with type 2 diabetes were regularly screened for both eGFR and albuminuria. An Australian cross-sectional study [16] found that among 90,550 patients with type 2 diabetes, only 44,394 (49.0 %) were appropriately screened or monitored. Rates in the present study, therefore, are plausible.

There are a number of potential reasons for this low screening rate. First, it may be the result of partial guideline inconsistencies. For example, the 2020 ADA guidelines [4] recommend only to screen for CKD in type 1 diabetes if diabetes duration is ≥ 5 years. This criterion is not met for at least some of the patients in the current dataset with a median diabetes duration of 11.9 years and an interquartile range of 2.7 to 25.0 years. Secondly, as multiple determinations of urinary albumin (at least two tests out of three need to be positive to arrive at a diagnosis of microalbuminuria) are necessary, the efforts for screening may be perceived to be high. Conversely, albuminuria tests are sometimes regarded as unreliable, especially if only performed once. Thirdly, not every physician’s office is able to collect urinary samples, which may appear unhygienic and, thus, not desirable. As a potential limitation, we did not explore whether a twice-annual monitoring of kidney function is actually performed in patients with advanced disease (urinary albumin > 30 mg/g creatinine and/or eGFR < 60 mL/min/1.73 m^2^). Some patients counted as screened in our analysis, would not be classified based on a requirement for two annual investigations as suggested by the ADA.

Finally, only about half of all patients in our database were screened based on both urinary albumin and creatinine. As opposed to the majority of guidelines, a determination of urinary albumin without normalization to creatinine appears to be frequent in clinical practice. This is of concern as a simple urinary dipstick test has a lower sensitivity and higher false-discovery rate compared to UACR-based screening [17, 18]. It has been previously described that there is no standardised method of collection and measurement of urinary albumin and creatinine and there are concerns about test reliability, intra-individual variability based on body position, activity and temperature [19, 20].

### Blood glucose control and treatment

Only 43.5 % of patients with type 2 diabetes reached the ESC/EASD [3] recommended HbA1c treatment target of < 7.0 %. Glucose control in type 2 diabetes is generally a matter of debate and a patient-centred approach suggests that individual blood glucose targets should be pursued. Variables that influence the actual HbA1c target are patient age, hypoglycaemic risk, and the comorbidity and co-medication burden of patients. While the 2020 ADA guidelines recommend no specific treatment target [4], 2020 KDIGO recommends an individualised HbA1c target range between < 6.5 % and < 8.0 % with higher targets tolerated for patients with present/severe macrovascular complications, many comorbidities and high hypoglycaemia risk [14]. Moreover, KDIGO recommends looser HbA1c targets in patients with severe CKD, which is at odds with the ESC/EASD recommendation [3]. This is in principal alignment with the recent German recommendations which pursue a corridor of 6.5–7.5 % to prevent CKD with the upper values recommended for patients with macroangiopathy. To prevent progression, an HbA1c value of 7 % or less is requested [18].

Less than 10 % of patients with type 2 diabetes received the two treatment options (SGLT-2i and/or GLP1-RAs) recommended for the reduction of renal endpoints by ESC/EASD [3]. The 2020 ADA guidelines [4] is slightly more specific than the ESC/EASD guidelines as to the use of SGLT-2i [3]. It recommends SGLT-2i use in those patients with type 2 diabetes with an eGFR ≥ 30 mL/min/1.73 m^2^ and urinary albumin > 30 mg/g creatinine, particularly in those with urinary albumin > 300 mg/g creatinine. KDIGO 2020 recommends SGLT-2i in patients with an eGFR ≥ 30 mL/min/1.73 m^2^ on the basis of metformin and GLP-1 RAs or in patients who are unable to tolerate these aforementioned medications [14]. Actual rates in DIVE/DPV were, however, very low with ~ 9 % and ~ 6 % of the patients with type 2 diabetes receiving SGLT-2i and GLP-1 Ras, respectively. These numbers have to be interpreted in light of their more recent market introduction, limitations by the approved indication (start of treatment until eGFR ≥ 60, stop treatment from eGFR < 45 ml/min/1.73 m^2^), the only recent recommendation in guidelines (using the 2020 guidelines as a reference) and the lack of documentation for potential reasons for their non-use.

About 27.2 % of patients with type 1 diabetes in DIVE/DPV reached a target HbA1c < 7.0 and 1.5 % (SGLT-2i)/0.6 % (GLP-1 RAs) received guideline-preferred anti-diabetic drugs as defined by the ESC/EASD [3]. While HbA1c treatment targets are no less stringent in type 1 than type 2 diabetes, the value of SGLT-2i/GLP-1 RAs in type 1 diabetes is less well documented. In 2019, the European Medicines Agency (EMA) approved a first SGLT-2i and a first dual SGLT-1/-2i to improve glycaemic control, as an adjunctive treatment to insulin in people with type 1 diabetes and a BMI ≥ 27 kg/m^2^ [21]. Of note, these are not approved for patients with type 1 diabetes by the Food and Drug Administration (FDA). No GLP1-RAs are registered for use in type 1 diabetes and neither agency recommends their use in patients with CKD. As such, the low use rate in type 1 diabetes is not surprising, even more so as non-insulin antidiabetic treatment in type 1 diabetes is rare anyway.

### Hypertension control and treatment

Blood pressure was controlled for 41.1–68.4 % in patients in DIVE/DPV depending on diabetes type and age for which different targets have been defined [3]. These findings are supported by another German study, which showed that blood pressure in patients with diabetes is insufficiently managed [22]. The ESC/EASD guidelines are very specific in recommending SBP to 130 mmHg and < 130 mmHg if tolerated, but not < 120 mmHg in patients between 18 and 65 years and 130–139 mmHg in patients > 65 years. The 2020 ADA and KDIGO [4, 14] give no specific recommendations as to the target blood pressure pursued, but rather confine their recommendation to the use of RAAS-blocking agents (ACEI or ARB) like the ESC/EASD. We documented ACEi use in 24.0 % (type 1 diabetes) and 39.9 % (type 2 diabetes) in patients with microalbuminuria with higher rates seen in patients with macroalbuminuria/proteinuria (40.9 %/47.7 %) which is below the rates observed for patients with type 2 diabetes in a Finnish study (57.0 %) [15]. KDIGO also recommends titration of the RAAS-blocker to the highest approved dose that is tolerated. The dose (and the presence of LVH) was not documented in our dataset, which prevents us from judging adequacy of treatment in this respect.

### Areas in need of future study or ongoing research

Guidelines conform in demanding regular screening for CKD in patients with diabetes, tight glucose control with the use of SGLT-2i (and GLP-1 RAs), and tight blood pressure control using ACEi/ARBs in those with hypertension and any degree of albuminuria. Inconsistencies are observed when looking at specific recommendations. The degree of translation into clinical practice has room for further improvement, but is somewhat uncertain because of the lack of specific data on the presence of drug contraindications, drug interactions and concomitant disease.

Based on our analysis we believe that (1) partial inconsistency between guidelines with respect to screening and treatment efforts prevents clinical practice to catch up and fully comply with these recommendations. (2) Further research is needed into the use of antidiabetic drugs and their potential benefits in CKD patients with type 2 and potentially even more so in type 1 diabetes. (3) Clear-cut recommendations on the use of antihypertensive and renoprotective drugs is needed. These drugs are widely used irrespective of the presence of albuminuria, but need to be consistently used in those with renal disease. (4) Beneficial drugs are usually not titrated to a maximally tolerated and effective dose resulting in suboptimal effects on renal and overall cardiovascular endpoints. Conversely, over-diagnosis and over-treatment may be deleterious to patients and society from a health and cost perspective and we encourage individual treatment decisions for each patient to maximise the benefits of current (and future) diagnostic and therapeutic treatment options.

### Limitations

The main limitation is the retrospective nature of this study, using data from a registry. However, due to the number of patients enrolled in the registry, we can be confident that the data provides a true reflection of diabetes patients in Germany. In addition, the data quality is very much dependent on the information included in the registry by the patient’s prescribing physician. There may be reasons for justified non-compliance to treatment guidelines but this information is not captured in the registry. Finally, this is a single-country study – based on German data – and may not reflect guideline compliance in other countries.

## Conclusions

The data indicate that there is room for improvement in the care of patients with diabetes with respect to renal disease diagnosis and treatment. While specific and potentially clinically justified reasons for non-compliance exist, the data may serve well for a critical appraisal of clinical practice decisions.

## Data Availability

The data that support the findings of this study are available on reasonable request from the corresponding author. The data are not publicly available due to privacy or ethical restrictions.
